# Personality Traits, Anxiety, and Self-esteem in Patients Seeking Cosmetic Surgery in Mexico City

**DOI:** 10.1097/GOX.0000000000002381

**Published:** 2019-10-30

**Authors:** Estephania Del Aguila, Jorge R. Martínez, José L. Pablos, Marino Huánuco, Victor M. Encina, Ana L. Rhenals

**Affiliations:** From the * Department of Psychiatry, Clínica Emocia, Mexico City; † Department of Plastic Surgery, Clínica Perfect Surgery; Mexico City; ‡ Department of Physics and Mathematics, Instituto Tecnológico y de Estudios Superiores de Monterrey, Mexico City Campus; § Department of Dermatology, Clínica dermatológica Soprano Skin, Mexico City; ¶ Private practice; ‖ Department of Nutrition, Clínica Perfect Surgery, Mexico City.

## Abstract

There are reports on high psychological morbidity, mainly personality disorders, among people opting for cosmetic surgery. This study aims to describe the personality traits, level of self-esteem, and anxiety in women seeking cosmetic surgery with body contouring, such as liposculpture or lipoabdominoplasty with or without placement of breast implants.

**Methods:**

Subjects were 87 women between 18 and 60 years of age in Mexico City who went to a private clinic with the intention of undergoing cosmetic surgery with body contouring. A psychiatric interview was performed using three scales: the Salamanca questionnaire for screening for personality disorders, the Hamilton Anxiety Rating Scale, and the Rosenberg Self Esteem Scale.

**Results:**

The average age was 31 years, with 35% of participants having previously undergone body-contouring surgery. Regarding personality, the patients did not show a psychopathology level for diagnosing a personality disorder. Regarding anxiety, 92% of the patients showed an average level of anxiety. High level of self-esteem was exhibited by 81 participants (93.15%), and six participants (6.9%) exhibited average self-esteem. Notably, no participant exhibited low self-esteem.

**Conclusions:**

The level of psychopathology of the patients was lower than expected, the self-esteem was not affected prior to the surgical procedure, and the level of anxiety did not cause dysfunction.

## INTRODUCTION

There are reports of high psychological morbidity among people opting for a cosmetic surgery.^1–9^ Up to 71% of people with personality disorders, mainly borderline-type emotional instability disorder,^2,10,11^ seek aesthetic surgical procedures with autolytic aims or unrealistic expectations about the outcomes^2,7^; people with personality disorders are described as demanding and irritable.^12^ Generalized anxiety disorder is one of the most frequent disorders in the Mexican population, with a prevalence of 14.3%, mainly in women between 40 and 50 years of age,^13^ with higher anxiety in younger women before a surgical procedure.^14–17^ The majority of patients are satisfied with the results and report an improvement in the quality of life after cosmetic surgery.^2,18–21^

A positive change in physical appearance has a positive psychological effect that improves self-confidence and self-esteem..^2,17^ Satisfaction with cosmetic surgery results has also been related to age, since older patients have more realistic expectations and are more satisfied with the results than younger patients..^21^ Some studies have found that self-esteem is not a determining component in the motivation for cosmetic surgery.^22^

This study aims to describe personality traits, level of self-esteem, and anxiety in women undergoing cosmetic surgery with body contouring, such as liposculpture or lipoabdominoplasty with or without placement of breast implants.

## PATIENTS AND METHODS

### Patients and Procedure

Subjects were female patients who opted for cosmetic and reconstructive surgery in a private practice in Mexico City between May 2016 and May 2017 with the intention of having a body-contouring cosmetic surgery. The investigation procedure was explained to them, and an informed consent was also obtained. The inclusion criteria were being a woman between 18 and 60 years of age, giving informed consent to participate in the study, and knowing how to read and write. When they met these criteria and consented to participate in the study, they were asked for their sociodemographic data. A single psychiatric interview was performed, and three scales were applied.

### Scales

#### Salamanca Questionnaire for Screening for Personality Disorder

It is an interviewer-administered questionnaire with 22 items, two for each personality disorder, ie, 11 personality traits/disorders that are subdivided into three groups: group A (paranoid, schizoid, schizotypal), group B (antisocial, narcissistic, histrionic, emotional instability borderline subtype, emotional instability impulsive subtype), and group C (anankastic, dependent, and anxious).^23,24^ These subdivisions have been used in different classifications for personality disorders, such as the International Statistical Classification of Diseases and Related Health Problems 10th Revision, Diagnostic and Statistical Manual of Mental Disorders (DSM IV), DSM IV-TR, and DSM 5, on which this scale was based and which has thus grouped personality traits and disorders based on their similarity.^24,25^ Each disorder trait has a score range from zero to six^24^; in this study, a disorder was considered present with a score of six, and a marked trait was considered present with three to five points, while a patient “without disorder” was considered with zero to two points.

The Salamanca questionnaire was developed at the Faculty of Medicine of the University of Salamanca, as part of a doctoral thesis, reporting an average total score of 1.35 and corroborating its clinical utility.^24^

Personality disorders are an enduring pattern of inner experience and behavior that deviate significantly from expectations of the individual’s culture, being inflexible and dominant in a variety of personal and social situations, causing deterioration in their functioning.^23^

The main characteristics corresponding to different personality traits/disorders assessed by the scale are described hereunder.^24–28^

### Hamilton Anxiety Rating Scale

An interviewer administered a scale that aims at determining the severity of symptoms of anxiety. It consisted of 14 items, 13 qualifying the symptoms of psychological anxiety (psychological malaise) and somatic anxiety (anxiety-related physical symptoms), and one on the behavior during the interview. Its internal consistency is 0.79–0.8, and test-retest reliability is 0.96.^29,30^ Each item has a score from zero to four (zero = no symptoms, and four = maximum intensity), with a total score range from 0 to 56 resulting in an average, moderate to average, and moderate to severe severity score.^29,30^

### Rosenberg Self-esteem Scale

It is a self-administered scale with 10 items. Cronbach’s α reliability is equal to 0.754, between 0.46 and 0.67 (*P* < 0.001). Internal consistency of each factor is expressed through α, Cronbach’s α 0.786–0.705. This scale meets the criteria for validity and reliability of a quality instrument for measuring self-esteem in the Latino population.^31^

### Psychiatric Assessment

A psychiatric evaluation was done to rule out a personality disorder, anxiety disorder, or problems in self-esteem. The evaluation consisted of a Semi-structured Psychiatric Interview and Free Forum with emphasis on psychopathological exploration, complemented with application of the scales mentioned above.

### Statistical Analysis

For the variable of age, its descriptive statistics were obtained: mean, mode, SD, and range. For the variables of occupation, religious faith, education, nationality, drug addictions, type of surgery planned, history of depression, history of cosmetic surgery, history of psychological treatment, and history of taking antidepressants, the distribution of observed and relative frequencies was obtained. The medians of age were compared between occupational types using the Kruskal-Wallis nonparametric test; previously, Shapiro-Wilk’s test of homogeneity of variances and Kolmogorov’s test were conducted.^32^ For the Hamilton Anxiety Rating Scale, the Rosenberg Self-Esteem Scale, and the Salamanca personality disorder scale, their reliability was assessed.^33^

For the Salamanca scale, which consists of 11 reagents, its validity and consistency, corresponding to the instruments of this type, were also determined. Regarding validity, a factor analysis was conducted. The scale is an instrument that measures the latent structure of the measurements of a psycho-social construct, such as the 11 personality traits. This procedure consists of finding sets of variables that characterize the latent structure under study. The method of principal components was applied to the correlation matrix and Kaiser’s varimax rotation of factors.^34^

The distribution table of each personality trait and level of presentation was obtained and classified into no disorder, marked trait, and disorder. The table was tested for independence of the rows and columns with Pearson’s correlation analysis. A Microsoft Excel database was developed with the data obtained. Processing for descriptive statistics, Cronbach’s α, and factor analysis were performed using the Statistical Package for the Social Sciences software package, version 21. Correspondence analysis was carried out using the package FactorMineR^35^ and factorextra^36^ in the statistical computer program R, version 3.3.1^37^ (Tables 1–3).

**Table 1.** Main Personality Trait/Disorder Characteristics

Personality Trait/Disorder	Main Characteristics
Group A	
Paranoid	Distrust or extreme suspicion
Schizoid	Detachment in social relationships Solitary Emotionally cold
Schizotypal	Social and interpersonal deficiencies Strange beliefs Strange or inappropriate behavior Magical thinking
Group B	
Histrionic	Excessive emotionality Attention-seeking Use of physical appearance to draw attention
Antisocial	Inattention to and infringement on the rights of others Breach of social norms Irresponsibility Absence of remorse
Narcissistic	Dominant pattern of grandeur Need for admiration Lack of empathy Feelings of grandeur and self-importance Arrogant attitudes Feelings of envy towards him/her or towards others
Borderline-type emotional instability	Instability of interpersonal relationships Desperate efforts to avoid abandonment Alteration of the self-image Chronic feeling of emptiness Sexually seductive or provocative behavior Suicide threats Self-harm
Impulsive-type emotional instability	Instability of interpersonal relationships Explosive personality Impulsivity
Group C	
Anankastic	Perfectionism Excessive devotion to work Excessive punctuality Rigidity and stubbornness
Dependent	Excessive need to be taken care of Submissive behavior and exaggerated attachment
Anxious	General pattern of social inhibition Feelings of inferiority Hypersensitivity to negative evaluation

**Table 2.** Sociodemographic Data, n = 87

	Category	N	Percentage
Marital status *	With partner	34	42.5
	No partner	46	57.5
Occupation *	Employed	39	48.8
	Independent	23	28.8
	Housewife	10	12.5
	Student	6	7.5
	Retired	1	1.3
	N/A	1	1.3
Education *	Primary	3	3.8
	Secondary	17	21.3
	High school	31	38.8
	Undergraduate	24	30.0
	Graduate	5	6.3
Religion *	Catholic	60	75
	Christian	6	7.5
	Jewish	1	
	N/A	13	16.3
Nationality	Mexican	85	2.3
	Colombian	2	97.7
Drug abuse	Tobacco	18	22.5
	Alcohol	5	6.3
	Marijuana	2	2.5
	Al + tab + mari	1	1.3
	Cocaine	2	2.5
	Amphetamines	1	1.3
	N/A	49	61.3
History of depression	Yes	39	45
	No	48	55
History of cosmetic surgery	Yes	30	35
	No	57	65
History of psychological treatment	Yes	28	32.5
	No	59	67.5
History of taking antidepressants	Yes	3	2.61
	No	85	97.39

* Only counted with information on 80 patients.

**Table 3.** Type of Cosmetic Surgeries Performed

Surgeries Performed	Frequency	Percentage
Breast implants	6	7.5
Liposculpture	23	28.8
Liposculpture and breast implant	24	30.0
Lipoabdominoplasty	13	16.3
Lipoabdominoplasty and breast implant	14	17.5
Total	80	100.0

## RESULTS

During the period from May 2016 to May 2017, a sample of 87 (n = 87) women with age ranging from 18 to 60 years was obtained, the mean being 31 years, and the SD being 8.9 years, whereas the mode corresponded to 26 years. Their occupations were as follows: 48.8% employed women (waitresses, models, or personal assistants), 28.8% self-employed women (itinerant traders or independent professionals), 12.5% housewives, 7.5% students, 1.3% retired, and 1.3% without occupation.

On analysis of the ages by occupation group, the Shapiro-Wilk and Kolmogorov tests showed that this variable does not follow a normal distribution (*P* = 0.01); the comparison of the medians of age between the occupational groups with the Kruskal-Wallis test showed no significant differences (*P* = 0.28).

Regarding religion, the most common was the Catholicism (75%), followed by Christianity (7.5%) and a single case of Judaism. No religion was reported in 16.3% of women. All the patients were Mexican, except for two Colombians, and 42.5% reported living in cohabitation, and 57.5% reported not having a cohabiting partner. The level of education was as follows: 38.8% high school, 30% undergraduate, and 6.3% graduate.

When the psychiatric evaluation was carried out, the following data were obtained. At least one episode of depression before surgery was reported by 45% of participants. Of these, only three reported having received antidepressant treatment, depression being independent of education (*P* > 0.05). Only 32.5% of patients at some point turned to psychological care. Analysis of the relationship between history of psychological treatment and education showed that 54.2% of the patients with a bachelor’s degree had sought treatment, while only 22.6% of those with the high school level did so (*P* = 0.05).

Most of them did not present serious substance abuse. Out of all, 22.5% participants met the criteria for harmful tobacco use followed by alcohol use (alone or combined with tobacco and marijuana; 9.1%) and 2.5% for harmful use of cocaine and/or amphetamines.

Body contouring surgery has been previously done in 35% of patients. In a search for an association between history of depression and previous cosmetic surgery, Fisher’s exact test turned out to be nonsignificant (*P* = 0.482).

Some psychotherapeutic and/or pharmacological treatment was suggested to 28.7% of participants at the end of the interview; the types of treatment were psychotherapeutic attention for problem solving, control of emotions, pharmacological treatment for insomnia, anxiety, and/or depression.

### Salamanca Questionnaire for Screening for Personality Disorder

**Fig. 1.**
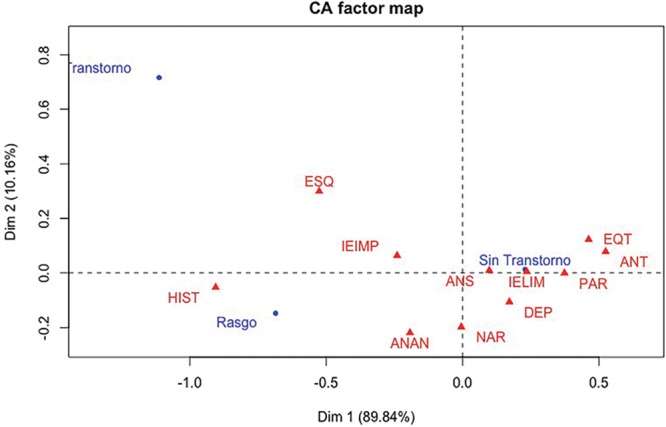
Nine of the 11 personality types are identified by not showing a personality disorder. This means that the patients showed the traits IE IMP, ANAN, ANS, NAR, IE LIM, DEP, PAR, ANT, and EQT without being marked traits or corresponding to a disorder. Eleven personality traits/disorders that are subdivided into 3 groups: group A [paranoid (PAR), schizoid (ESQ), schizotypal (EQT)], group B [antisocial (ANT), narcissistic (NAR), histrionic (HIS), emotional instability borderline subtype (IE LIM), emotional instability impulsive subtype (E IMP)], and group C [anankastic (ANAN), dependent (DEP), and anxious (ANS)].

The reliability analysis of this scale showed Cronbach’s α had a value of 0.789 (mean 0.720 ± 0.469), and minimum and maximum values of 0.023 and 1.678, respectively. Regarding the validity of the Salamanca scale, factor analysis shows that four factors are necessary to explain only 61.93% of the total variability of the 11 traits/personality disorders. Table 4 shows the factors and personality traits that characterize each of them. If the traits have coefficients greater than absolute value of 0.5, it can be considered that anxiety and dependence do not manifest in any factor, allowing for the interpretation that the items used do not detect these traits. On the other hand, it is important to note that the traits that characterize each of the four factors are consistent with the distribution of international classifications.^22–24^ When this questionnaire was applied, the distribution of traits was obtained to determine the level of the personality trait (without disorder, marked trait, and disorder), which is presented in Table 5. The test of independence of level and personality types yielded Pearson’s chi-square statistic value of 185.33 (*P* = 0.00001), which shows a high degree of dependence between these two variables. The analysis of correspondence between disorders and personality traits in Table 5 showed that the first axis explains 89.84% of the total variance, while the second axis contributes to another 10.16%. Figure 1 shows the correspondence of the 11 personality types and the three trait/disorder levels.

**Table 4.** Coefficients of the Factors of the Salamanca Scale

Trait/Disorder	Factors			
	1	2	3	4
Paranoid			0.708	
Schizoid			0.749	
Schizotypal		0.768		
Histrionic	0.662			
Antisocial				−0.735
Narcissistic				0.511
Impulsive	0.746			
Borderline	0.836			
Anankastic		0.762		
Dependent*				
Anxious*				

Method of extraction: Principal component analysis.

Method of rotation: Varimax normalization with Kaiser.

a. The rotation has converged in seven iterations.

* If we consider the features whose coefficients are greater in absolute value of 0.5, it can be seen that anxious and dependent do not appear in any factor, which can be interpreted as the items used do not detect these features.

**Table 5.** Frequency of Personality Traits and Their Level of Presentation

Level	PAR	ESQ	EQT	HIS	ANT	NAR	IE IMP	IE LIM	ANAN	DEP	ANS
Disorder	0	10	1	8	0	0	5	1	1	0	2
Marked trait	7	28	2	46	1	22	24	11	28	15	15
No disorder	80	49	84	33	86	65	58	75	58	72	70

Depressive (DEP), anxious (ANS), Unstable Impulse (IE IMP), Unstable Borderline (IE LIM), Schizotypal (EQT), Histrionic (HIS), Narcissistic (NAR), Schizoid (ESQ), Anankastic (ANAN), Paranoid (PAR), and Antisocial (ANT).

The second level of trait/disorder intensity, ie, marked trait, corresponds to the histrionic trait with 46–87 patients belonging to this group. It is interesting to note that no personality type was associated with the disorder, which means that patients did not show a psychopathology level for diagnosing a personality disorder.

### Hamilton Anxiety Rating Scale

The internal reliability coefficient, Cronbach’s α, was equal to 0.61, with mean value of 0.356 ± 0.264 and minimum and maximum values of 0.057 and 0.885, respectively. Seven participants received a score of zero, which is indicative of a person with no presence of anxiety. This scale assigns the average anxiety category to people with a score below 17. Therefore, 92% of the patients showed an average level of anxiety.

### Rosenberg Self-esteem Scale

Cronbach’s α was equal to 0.704, with a mean value of 3.500 ± 0.268 and maximum and minimum values of 3.057 and 3.770, respectively. In this study, 81 patient (93.15%) exhibited a high level of self-esteem, and six (6.9%) exhibited an average level of self-esteem. Notably, no participant exhibited low self-esteem.

## DISCUSSION

It was observed that women who came in search of body-contouring cosmetic surgical treatment in this practice, 35% of whom had previously undergone a cosmetic surgery, were mostly single, of an economically productive age (around 29 to 31 years), with work experience as subordinates, and with schooling between high school and undergraduate.

About mental health history, 45% reported of having had an episode of depression during their lifetime, but only 32% reported receiving psychological attention and 3.8% having taken some antidepressant drug treatment.

Regarding personality, 31% obtained a score of 6 for a disorder, the highest percentages being those of schizoid type (11.49%), histrionic (9.19%), and emotional instability (borderline) (6.88%).

The most frequently presented marked trait was histrionic (52.87%). That is having marked histrionic-type traits encouraged the search for body-contouring cosmetic surgery more than borderline personality disorder did. The histrionic traits are characterized by the search for attention, frequently using the physical aspect to attract it.^22^ This explains why the marked traits were mostly presented. Therefore, the search for surgery was motivated by a desire for attention and approval from society but not for internal satisfaction that would have affected their self-esteem.

Most of them did not show alterations in their self-esteem, and 93.15% showed high self-esteem. Some studies have reported that a positive change in physical appearance has a positive psychological effect, improving self-confidence and self-esteem.^2,20^ In this study, self-esteem was at adequate levels, and thus low self-esteem was not the incentive for seeking body-contouring surgery. The motivators for undergoing cosmetic surgery were not conditioned by the search to improve self-esteem. On the contrary, having a high self-esteem was a characteristic of participants opting for this type of surgery.

Out of all participants, 92% had a score corresponding to average anxiety, which does not cause dysfunction in everyday life. Thus, this did not affect their decision, behavior in the surgical event, or postsurgical recovery.

These results make us think about the great influence that external factors from society have on women and the value given to female body image, since these were the main incentive for body-contouring cosmetic surgery. It is important to conduct a longitudinal follow-up study after surgery and observe if there are changes in self-esteem. Regarding personality, we believe that in the future, it will be convenient to look for scales with optimal properties of specificity, consistency, and reliability to describe personality traits of this type of patients.

### Strengths

Firstly, we have obtained a random sample where patients have consented to a psychiatric interview, despite this not being their reason to go to the private plastic surgery practice. Secondly, the sample may be an example of private consultation in plastic surgery for cosmetic and non-reconstructive purposes, with a population that can be considered healthy.

### Limitations

First of all, this is a cross-sectional study, which has not made it possible to see if there are changes after the surgical event. Secondly, there was no control group with a population coming from a public institution. Moreover, we used the scales that have a limited a number of items due to the time available for answering them. Additionally, the scale of personality traits with few items was used instead of the one with greater extension and consequently greater specificity.

## CONCLUSIONS

The patients’ level of psychopathology was lower than expected, there was a low number of patients with personality disorders, being those of schizoid type (11.49%), histrionic (9.19%), and emotional instability (borderline) (6.88%). The most frequently presented marked personality trait was histrionic (52.87%).

The motivators for undergoing cosmetic surgery were not conditioned by the search to improve self-esteem. On the contrary, having a high self-esteem was a characteristic of participants opting for this type of surgery.

Regarding anxiety, 92% of the patients showed an average level that was not a cause of dysfunction.

It is important to conduct a longitudinal follow-up study after surgery and observe if there are changes in self-esteem, anxiety, personality trait, and personality disorders.

## ACKNOWLEDGMENTS

All procedures performed in this study involving human participants were in accordance with the 1975 Helsinki Declaration. Informed consent form was delivered to all the patients by the investigators included in the study.
